# Supplementary material for: Multigene phylogeny of the scyphozoan jellyfish family Pelagiidae reveals that the common U.S. Atlantic sea nettle comprises two distinct species (Chrysaora quinquecirrha and C. chesapeakei)
Source: PeerJ. 2017 Oct 13;5:e3863. doi: 10.7717/peerj.3863 (PMC5642265; doi:10.7717/peerj.3863)
Supplement: Supplemental Information 1 — Collected samples were collected by the authors while museum specimens all came from the Smithsonian National Museum of Natural History. Sites were categorized between coastal and estuarine regions based on geography, knowledge of regions and average salinity where available from data buoys, but deemed coastal if in doubt. *For collected regions, some specimens were sequenced for mitochondrial 16S to assign to a species/clade in Fig. 5. [file peerj-05-3863-s001.docx]

**Table S1**: Geographic source regions of samples used for morphological analyses in this study. Collected samples were collected by the authors while museum specimens all came from the Smithsonian National Museum of Natural History. Sites were categorized between coastal and estuarine regions based on geography, knowledge of regions and average salinity where available from data buoys, but deemed coastal if in doubt. *For collected regions, some specimens were sequenced for mitochondrial *16S* to assign to a species/clade in Figure 5.

|  | **Museum** | **Latitude/** | **Water** | **Coastal/** | ***n*** | | |  |
| --- | --- | --- | --- | --- | --- | --- | --- | --- |
| **Site** | **ID** | **Longitude** | **Body** | **Estuarine** | **morphology** | **molecular*** | **Nematocyst measurements** | **Nematocyst**  **Counts** |
|  |  |  |  |  |  |  |  |  |
| *Collected Samples* |  |  |  |  |  |  |  |  |
| Charlestown Pond, RI (RI) | - | 41.37 N 71.63 W | Atlantic | Estuarine | 14 | 9 | - | - |
| Cape Henlopen, DE (CHP) | - | 38.80 N 75.11 W | Atlantic | Coastal | 5 | 5 | 3 | 3 |
| Rehoboth Bay, DE (RB) | - | 38.69 N 75.08 W | Atlantic | Estuarine | 16 | 9 | - | - |
| Gloucester Point, VA (GP) | - | 37.25 N 76.50 W | Atlantic | Estuarine | 12 | 7 | 2 | 2 |
| Charleston, SC (CST) | - | 32.79 N 79.91 W | Atlantic | Estuarine | 6 | 6 | 1 | 1 |
| Dauphin Island, AL (DI) | - | 30.24 N 88.08 W | Gulf of Mexico | Coastal | 3 | 2 | 1 | 1 |
|  |  |  |  |  |  |  |  |  |
| *Museum Samples* |  |  |  |  |  |  |  |  |
| Buzzard’s Bay, MA | USNM 24496 | - | Atlantic | Coastal | 1 | - | - | - |
| Charlestown Pond, RI | USNM 56758 | - | Atlantic | Estuarine | 5 | - | - | - |
| Cape Henlopen, DE | USNM 54511 | - | Atlantic | Coastal | 2 | - | 1 | 1 |
| Cape Henlopen, DE | USNM 56702 | - | Atlantic | Coastal | 1 | - | - | 1 |
| Arundel on the Bay, MD | USNM 53867 | - | Atlantic | Estuarine | 1 | - | - | - |
| Arundel on the Bay, MD | USNM 33121 | - | Atlantic | Estuarine | 6 | - | - | - |
| Chesapeake Beach, MD | USNM 55621 | - | Atlantic | Estuarine | 6 | - | - | - |
| Plum Point, MD | USNM 33456 | - | Atlantic | Estuarine | 4 | - | - | - |
| Plum Point, MD | USNM 33457 | - | Atlantic | Estuarine | 4 | - | 1 | - |
| Plum Point, MD | USNM 33458 | - | Atlantic | Estuarine | 3 | - | - | - |
| Drum Point, MD | USNM 53828 | - | Atlantic | Estuarine | 2 | - | - | - |
| Open Chesapeake Bay | USNM 56703 | 37.23 N 76.04 W | Atlantic | Estuarine | 2 | - | - | - |
| Open Chesapeake Bay | USNM 56704 | 37.23 N 76.04 W | Atlantic | Estuarine | 4 | - | 1 | - |
| Open Chesapeake Bay | USNM 54404 | 37.25 N 76.08 W | Atlantic | Estuarine | 1 | - | 1 | - |
| Tom’s Cove, VA (TC) | USNM 53860 | - | Atlantic | Coastal | 1 | - | 1 | - |
| Tom’s Cove, VA | USNM 53861 | - | Atlantic | Coastal | 1 | - | 1 | - |
| Orange Inlet, NC | USNM 57925 | - | Atlantic | Estuarine | 9 | - | 1 | 1 |
| Beaufort, NC | USNM 53870 | - | Atlantic | Estuarine | 3 | - | - | - |
| Alligator Harbor, FL | USNM 49733 | - | Gulf of Mexico | Coastal | 1 | - | - | - |
| Timbalier Bay, LA (TB) | USNM 53826 | - | Gulf of Mexico | Coastal | 2 | - | - | - |
| Louisiana | USNM 41255 | - | Gulf of Mexico | Unknown | 2 | - | - |  |
| Lake Pontchartrain, LA | USNM 54372 | - | Gulf of Mexico | Estuarine | 1 | - | - |  |
