# Supplementary material for: Multigene phylogeny of the scyphozoan jellyfish family Pelagiidae reveals that the common U.S. Atlantic sea nettle comprises two distinct species (Chrysaora quinquecirrha and C. chesapeakei)
Source: PeerJ. 2017 Oct 13;5:e3863. doi: 10.7717/peerj.3863 (PMC5642265; doi:10.7717/peerj.3863)
Supplement: Supplemental Information 2 [file peerj-05-3863-s002.docx]

**Table S2**: PCR primers employed in this study (A=PCR amplification; S=DNA Sequencing)

| **Region** | **Primer Name** | **Sequence (5’-3’)** | **Purpose** | **Reference** |
| --- | --- | --- | --- | --- |
|  |  |  |  |  |
| COI | KMBMT-71 | TGGTGCTTTTTCAGCTATGATTGG | A, S | This study |
|  | LCOjf | GGTCAACAAATCATAAAGATATTGGAAC | A, S | Dawson (2005b) |
|  | HCO2198 | TAAACTTCAGGGTGACCAAAAAATCA | A, S | Folmer et al. (1994) |
|  | KMBMT-103 | CAGGGTGACCAAAAAATCAAAA | S | This study |
|  |  |  |  |  |
| 16S | Primer-1 | TCGACTGTTTACCAAAAACATAGC | A, S | Bridge et al. (1992) |
|  | Primer-2 | ACGGAATGAACTCAAATCATGTAAG | A, S | Bridge et al. (1992) |
|  | 16S-L | GACTGTTTACCAAAAACATA | A, S | Ender & Schierwater (2003) |
|  | Aa_H16S_1541H | AGATTTTAATGGTCGAACAGAC | A, S | Bayha & Dawson (2010) |
|  | KMBMT-46 | AACGCCAATAGGGGGTGCAA | S | This study |
|  | KMBMT-47 | CAACCCTTAGGAGCTGCTGC | S | This study |
|  |  |  |  |  |
| 28S | Aa_L28S_21 | GAACRGCTCAAGCTTRAAATCT | A, S | Bayha & Dawson (2010) |
|  | Aa_H28S_1078 | GAAACTTCGGAGGGAACCAGCTAC | A, S | Bayha et al. (2010) |
|  | Aa_L28S_48 | GCTTGCAACAGCGAATTGTA | S | Bayha et al. (2010) |
|  | Aa_H28S_1039 | GTCTTTCGCCCCTATACCCA | S | Bayha et al. (2010) |
|  | Aa_L28S_260 | ATAGCGAACAAGTACCGTGA | S | Bayha et al. (2010) |
|  | Aa_H28S_775 | ACTTGCGCACATGTTAGACT | S | Bayha et al. (2010) |
