# Supplementary material for: Multigene phylogeny of the scyphozoan jellyfish family Pelagiidae reveals that the common U.S. Atlantic sea nettle comprises two distinct species (Chrysaora quinquecirrha and C. chesapeakei)
Source: PeerJ. 2017 Oct 13;5:e3863. doi: 10.7717/peerj.3863 (PMC5642265; doi:10.7717/peerj.3863)
Supplement: Supplemental Information 3 — Values below the diagonal are minimum pairwise genetic distances computed using the Kimura 2-parameter substitution model (Kimura, 1980) in MEGA 7.0.14 (Kumar, Stecher & Tamura, 2016). Values in bold represent maximum within clade divergences. Column/row numbers represent major species/clades: 1. Chrysaora achlyos, 2. C. africana, 3. C. chesapeakei, 4. Chrysaora c.f. chesapeakei, 5. C. chinensis, 6. C. colorata, 7. C. fulgida, 8. C. fuscescens, 9. C. hysoscella, 10. C. lactea, 11. C. melanaster, 12. C. pacifica, 13. C. plocamia, 14. C. quinquecirrha, 15. Chrysaora sp. 1, 16. Pelagia benovici, 17. P. noctiluca, 18. Sanderia malayensis, 19. Cyanea capillata. [file peerj-05-3863-s003.docx]

**Table S3**: Pairwise genetic distance matrix (*COI*) for major clades/species in the Pelagiidae. Values below the diagonal are minimum pairwise genetic distances computed using the Kimura 2-parameter substitution model (Kimura 1980) in MEGA 7.0.14 (Kumar, Stecher & Tamura 2016). Values in bold represent maximum within clade divergences. Column/row numbers represent major species /clades: 1. *Chrysaora achlyos*, 2. *C. africana*, 3. *C. chesapeakei*, 4. *Chrysaora* c.f. *chesapeakei*, 5. *C. chinensis*, 6. *C. colorata*, 7. *C. fulgida*, 8. *C. fuscescens*, 9. *C. hysoscella*, 10. *C. lactea*, 11. *C. melanaster*, 12. *C. pacifica*, 13. *C. plocamia*, 14. *C. quinquecirrha*, 15. *Chrysaora* sp. 1, 16. *Pelagia benovici*, 17. *P. noctiluca*, 18. *Sanderia malayensis,* 19*. Cyanea capillata*.

|  | 1 | 2 | 3 | 4 | 5 | 6 | 7 | 8 | 9 | 10 | 11 | 12 | 13 | 14 | 15 | 16 | 17 | 18 | 19 |
| --- | --- | --- | --- | --- | --- | --- | --- | --- | --- | --- | --- | --- | --- | --- | --- | --- | --- | --- | --- |
|  |  |  |  |  |  |  |  |  |  |  |  |  |  |  |  |  |  |  |  |
| 1 | - |  |  |  |  |  |  |  |  |  |  |  |  |  |  |  |  |  |  |
| 2 | 0.204 | **0.005** |  |  |  |  |  |  |  |  |  |  |  |  |  |  |  |  |  |
| 3 | 0.215 | 0.193 | **0.022** |  |  |  |  |  |  |  |  |  |  |  |  |  |  |  |  |
| 4 | 0.197 | 0.197 | 0.062 | **0.051** |  |  |  |  |  |  |  |  |  |  |  |  |  |  |  |
| 5 | 0.233 | 0.222 | 0.222 | 0.222 | **0.000** |  |  |  |  |  |  |  |  |  |  |  |  |  |  |
| 6 | 0.094 | 0.222 | 0.222 | 0.219 | 0.240 | **-** |  |  |  |  |  |  |  |  |  |  |  |  |  |
| 7 | 0.193 | 0.189 | 0.134 | 0.127 | 0.214 | 0.217 | **0.013** |  |  |  |  |  |  |  |  |  |  |  |  |
| 8 | 0.122 | 0.199 | 0.201 | 0.212 | 0.230 | 0.134 | 0.199 | **-** |  |  |  |  |  |  |  |  |  |  |  |
| 9 | 0.208 | 0.197 | 0.110 | 0.128 | 0.201 | 0.228 | 0.083 | 0.212 | **0.002** |  |  |  |  |  |  |  |  |  |  |
| 10 | 0.212 | 0.206 | 0.181 | 0.151 | 0.208 | 0.235 | 0.143 | 0.206 | 0.152 | **-** |  |  |  |  |  |  |  |  |  |
| 11 | 0.120 | 0.193 | 0.208 | 0.210 | 0.210 | 0.129 | 0.204 | 0.140 | 0.195 | 0.239 | **0.013** |  |  |  |  |  |  |  |  |
| 12 | 0.210 | 0.181 | 0.185 | 0.170 | 0.206 | 0.240 | 0.170 | 0.219 | 0.162 | 0.166 | 0.216 | **0.005** |  |  |  |  |  |  |  |
| 13 | 0.212 | 0.197 | 0.142 | 0.146 | 0.230 | 0.224 | 0.066 | 0.206 | 0.098 | 0.147 | 0.219 | 0.174 | **0.010** |  |  |  |  |  |  |
| 14 | 0.191 | 0.149 | 0.121 | 0.129 | 0.202 | 0.213 | 0.122 | 0.196 | 0.130 | 0.143 | 0.180 | 0.174 | 0.146 | **0.003** |  |  |  |  |  |
| 15 | 0.186 | 0.183 | 0.191 | 0.178 | 0.193 | 0.239 | 0.178 | 0.205 | 0.187 | 0.168 | 0.205 | 0.138 | 0.204 | 0.191 | **-** |  |  |  |  |
| 16 | 0.246 | 0.230 | 0.203 | 0.211 | 0.203 | 0.251 | 0.216 | 0.240 | 0.211 | 0.239 | 0.218 | 0.208 | 0.220 | 0.220 | 0.211 | **0.003** |  |  |  |
| 17 | 0.244 | 0.267 | 0.250 | 0.263 | 0.245 | 0.266 | 0.278 | 0.237 | 0.263 | 0.252 | 0.279 | 0.252 | 0.268 | 0.246 | 0.251 | 0.269 | **0.053** |  |  |
| 18 | 0.262 | 0.217 | 0.228 | 0.228 | 0.232 | 0.282 | 0.219 | 0.248 | 0.224 | 0.233 | 0.273 | 0.191 | 0.210 | 0.212 | 0.208 | 0.142 | 0.251 | **-** |  |
| 19 | 0.250 | 0.238 | 0.228 | 0.198 | 0.212 | 0.250 | 0.230 | 0.217 | 0.223 | 0.227 | 0.232 | 0.229 | 0.217 | 0.206 | 0.244 | 0.229 | 0.275 | 0.237 | **-** |
