# Supplementary figures and images for: Multigene phylogeny of the scyphozoan jellyfish family Pelagiidae reveals that the common U.S. Atlantic sea nettle comprises two distinct species (Chrysaora quinquecirrha and C. chesapeakei)
Source: PeerJ. 2017 Oct 13;5:e3863. doi: 10.7717/peerj.3863 (PMC5642265; doi:10.7717/peerj.3863)

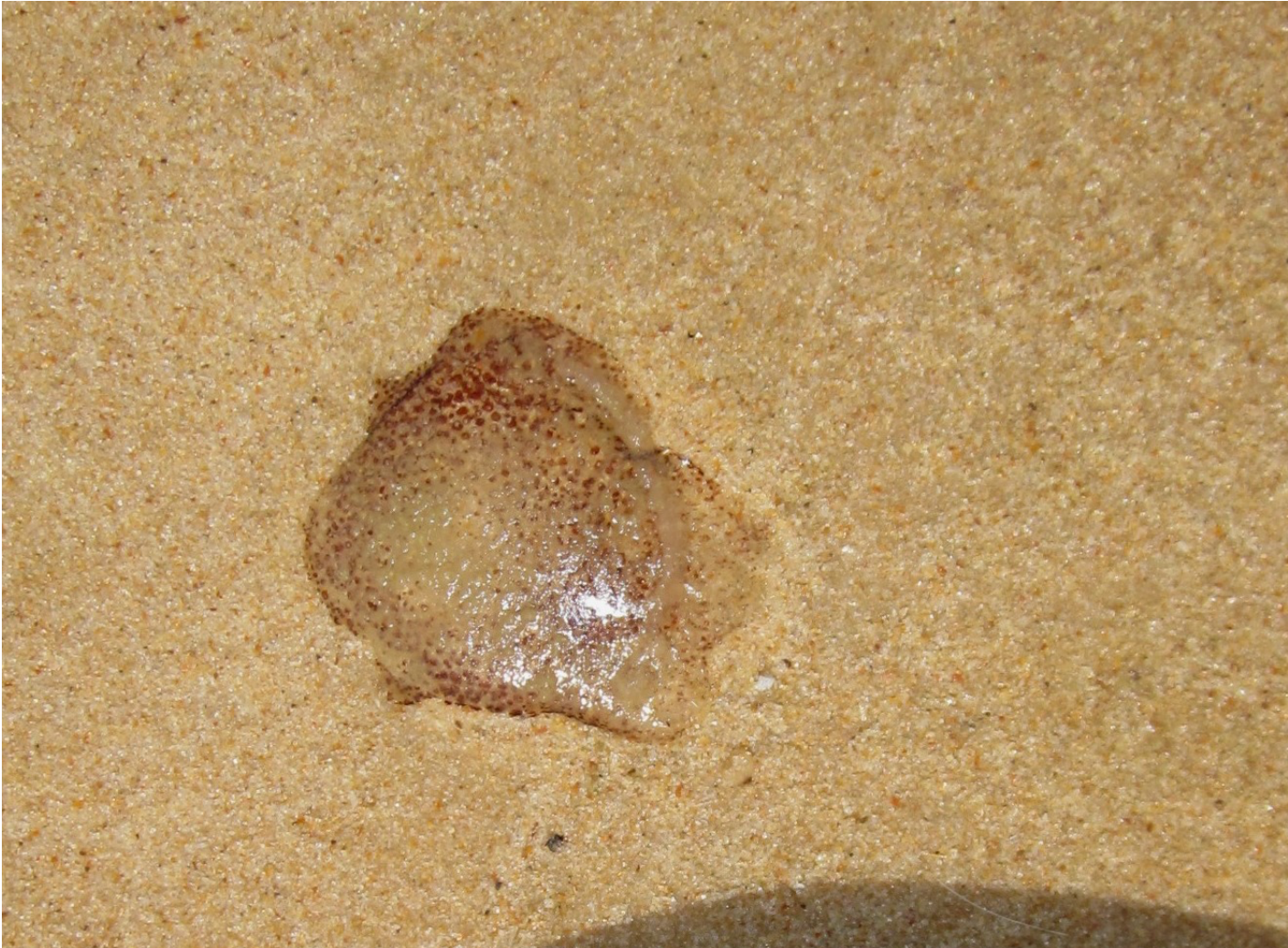

Supplement: Supplemental Information 6 — Photograph of unknown pelagiid jellyfish from Dakar, Senegal ultimately assigned to the species Mawia benovici based on DNA sequence data (28S and COI). The jellyfish bell was approximately 5–6 cm. Photograph courtesy of Lucy Keith-Diagne. [file peerj-05-3863-s006.pdf]

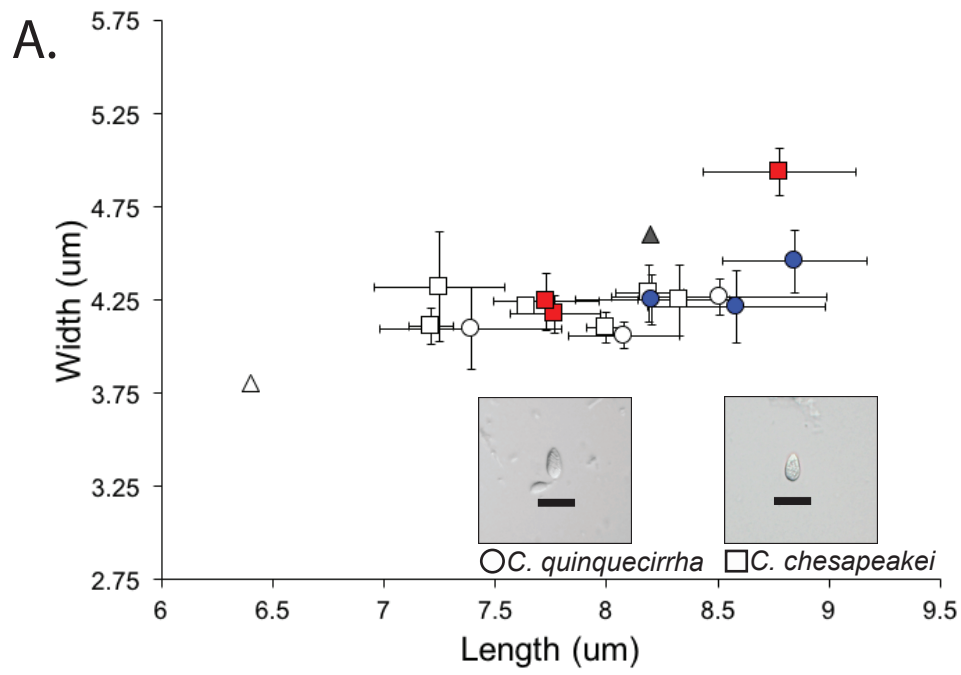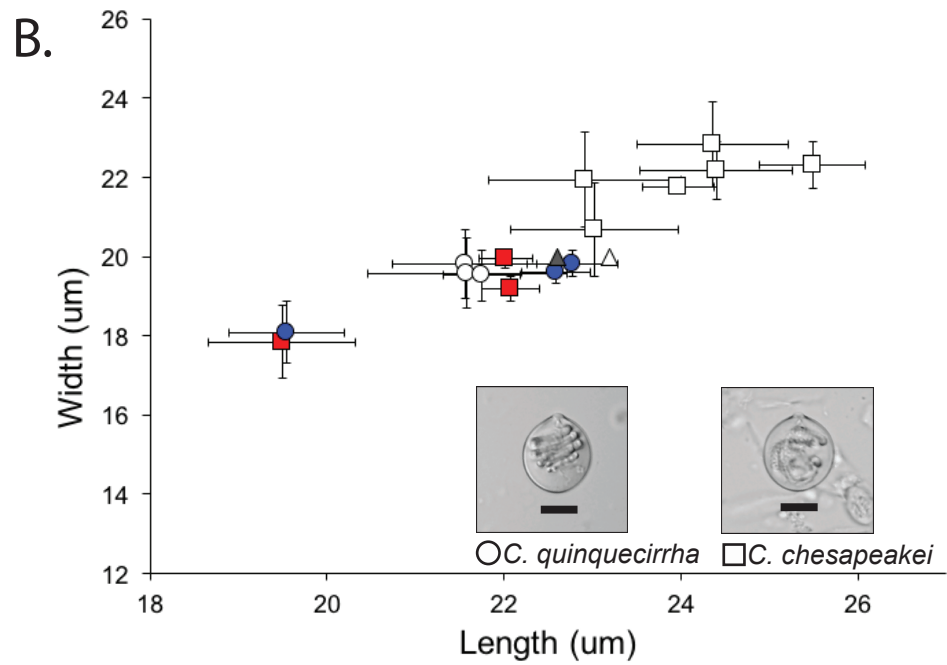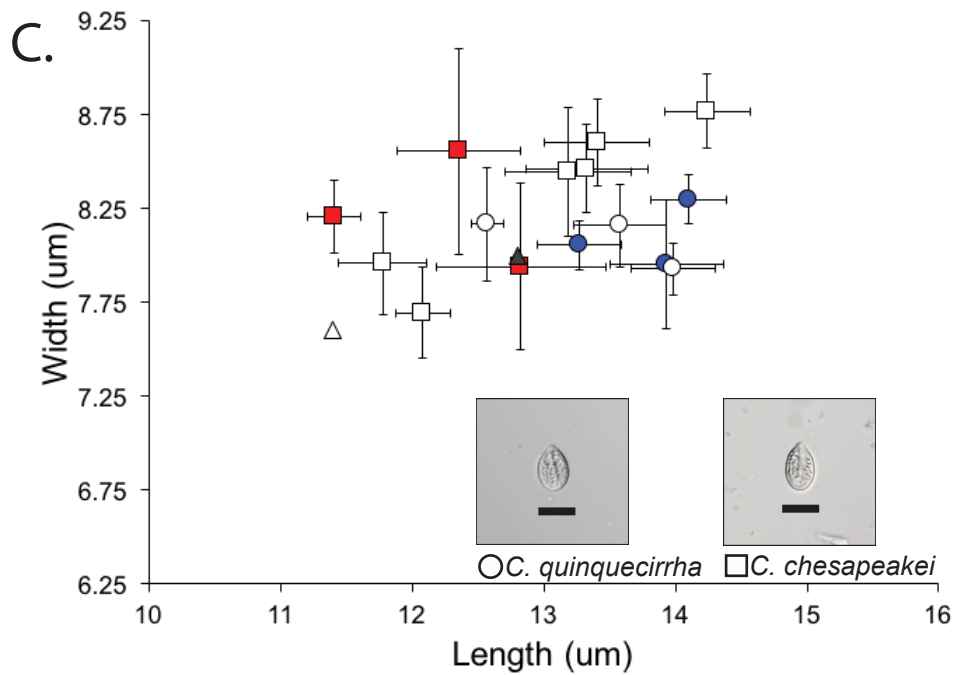

Supplement: Supplemental Information 7 — Average size measurements based on 10 nematocysts per individual (length vs. width) for nematocysts: A) a-isorhizas; B) O-isorhizas; C) heterotrichous microbasic birhopaloids. Error bars represent standard deviation values. Squares represent nematocysts from estuarine Atlantic and Gulf of Mexico medusae (C. chesapeakei), while circles represent nematocysts from coastal Atlantic medusae (C. quinquecirrha). All animals with 16S sequences matching the C. chesapeakei clade appear in red, while those whose sequences matched the C. quinquecirrha clade appear in blue. Triangles represent average values from Papenfuss (1936) for morphs identified as Dactylometra quinquecirrha (gray) or Dactylometra quinquecirrha var. chesapeakei (white). Nematocyst examples are to the right of each graph. All nematocysts are of average size for the nematocyst type and species. Photographs have been resized so that all error bars are the same size on the page to allow size comparisons. [file peerj-05-3863-s007.pdf]

A.

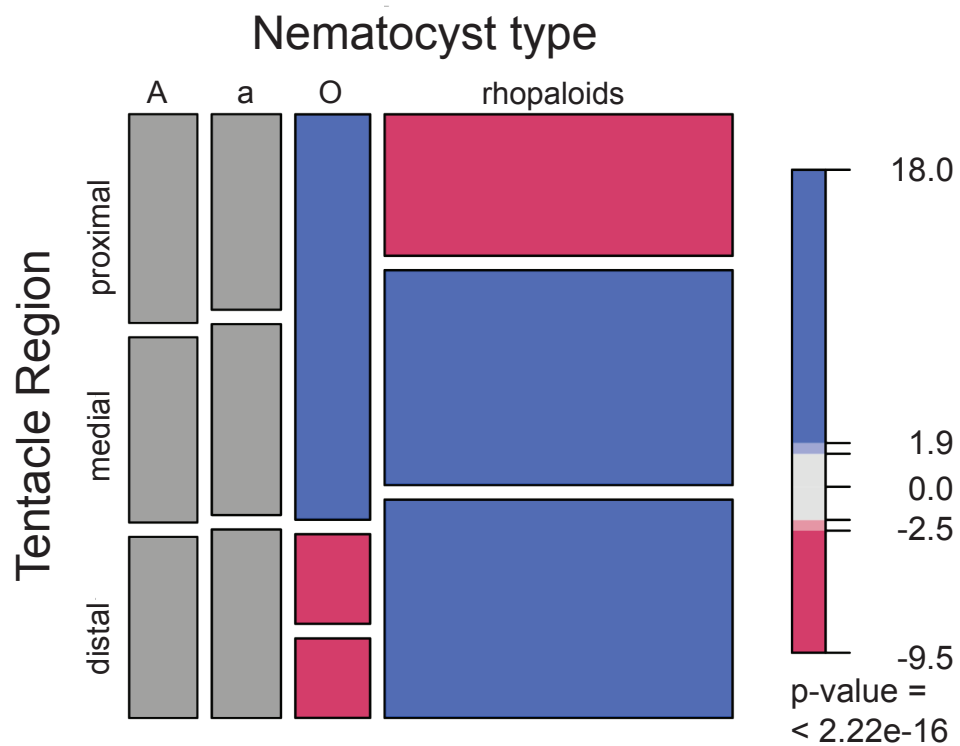

B.

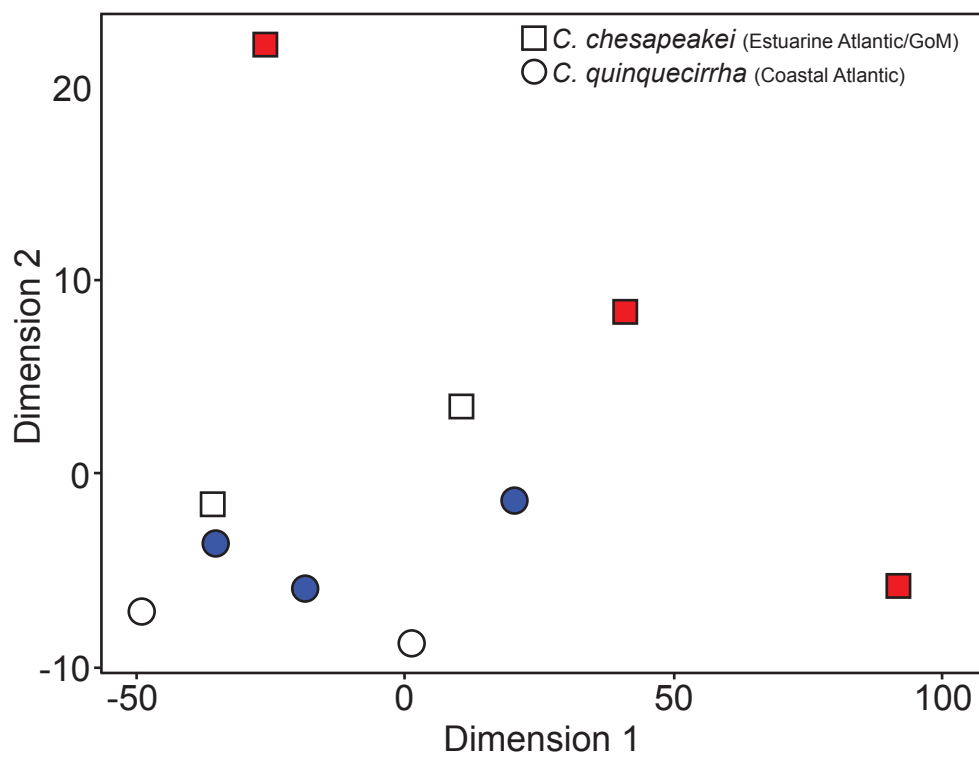

Supplement: Supplemental Information 8 — A) Mosaic plot showing the relative proportions of nematocyst types in distal, medial and proximal tentacle regions. O-isorhiza and birhopaloid nematocysts vary markedly in abundance across regions. Plot drawn using R package vcd (Meyer, Zeileis & Hornik, 2016). Proportions of nematocysts types vary significantly across tentacle regions; shading indicates significant departures from expected values (red = negative residuals, blue = positive residuals). B) Non-metric multidimensional scaling of similarities in overall (proximal, medial and distal regions) proportions of all four nematocyst types. Squares represent nematocysts from estuarine Atlantic and Gulf of Mexico medusae, while circles represent nematocysts from coastal Atlantic medusae. All animals with 16S sequences matching the C. chesapeakei clade appear in red, while those whose sequences matched the C. quinquecirrha clade appear in blue. [file peerj-05-3863-s008.pdf]
